# Prosociality and Personality: Perceived Efficacy of Behaviors Mediates Relationships between Personality and Self-Reported Climate Change Mitigation Behavior

**DOI:** 10.3390/ijerph20043637

**Published:** 2023-02-18

**Authors:** John B. Nezlek, Marzena Cypryańska

**Affiliations:** 1 Center for Climate Action and Social Transformations, Institute of Psychology, SWPS University of Social Sciences and Humanities, ul. Chodakowska 19/31, 03-815 Warsaw, Poland; 2 Department of Psychological Sciences, College of William & Mary, Box 8795, Williamsburg, VA 23185, USA

**Keywords:** personality, climate change mitigation behavior, perceived efficacy, environmental psychology

## Abstract

The included studies examined the relationship between climate change mitigation behavior (CCB) and personality. In Study 1, 1089 US collegians completed a measure of the Big Five and indicated how often they engaged in five CCBs. Engaging in each CCB was regressed on the Big Five. These analyses found openness was positively related to all five CCBs, neuroticism was positively related to four of five CCBs, and extraversion was positively related to three CCBs. In Study 2, 1688 US collegians completed the same measures as in Study 1 with two additional CCBs. They also indicated how efficacious they thought each CCB was. Each CCB was regressed on the Big Five. These results largely replicated those of Study 1 and also found that conscientiousness was positively related to five of seven CCBs. Mediational analyses found that all relationships between personality factors and CCB were mediated by the perceived efficacy of the CCB. The present results suggest that efforts to increase climate change mitigation behavior need to take into account the perceived efficacy of such behaviors.

## 1. Introduction

There is a broad scientific consensus that climate change represents an existential threat to humanity and life on Earth. If present trends in climate change are not reversed, life as it now exists on Earth will change dramatically. Species will disappear, arable land will no longer be arable, and the seas will become too warm to support the diversity of life they now support [1].

This paper is part of a special issue devoted to relationships between personality and well-being, with well-being defined broadly to include prosocial behaviors. We believe that engaging in climate change mitigation behavior (CCB), i.e., doing things to avert the catastrophe described above, is clearly prosocial, and as such, a paper about relationships between personality and CCB falls within the scope of the special issue. 

Fighting climate change is an example of what Nezlek [2] defined as *ideological prosociality*: “concerns people’s thoughts, beliefs, and behaviors that are intended to benefit others collectively, such as a concern for human rights, social equality, environmental quality, and so forth,” which contrasts with *interpersonal prosociality*: “concerns people’s thoughts, beliefs, and behaviors that are intended to benefit people directly, such as helping, providing social support, and so forth” (p. 1). In an analysis of Wave 8 of the European Social Survey, Nezlek found that although measures of interpersonal and ideological prosocial values were correlated (0.28), ideologically prosocial values were positively related to pro-environmental attitudes and advocating pro-environmental policies, whereas interpersonal prosocial values were not related to such attitudes and advocacy. As Nezlek noted, most of the existing research on prosociality has examined interpersonal prosociality.

We decided to examine the relationship between CCB and personality in terms of the traits that comprise the Big Five or Five Factor Model of personality. These are considered by many to represent the “building blocks” of personality and, by extension, have been assumed to be important influences on people’s thoughts, feelings, and behaviors. Consistent with this assumption, personality traits have been found to be manifested in various behaviors [3], including behaviors intended to mitigate climate change. The present studies were designed to complement and extend our understanding of the relationships between personality and CCB by examining how the perceived effectiveness (efficacy) of CCB mediated relationships between personality and CCB.

### 1.1. Climate Change Mitigation Behavior and Personality

A recent meta-analysis of relationships between personality and pro-environmental behavior (PEB) found that agreeableness, conscientiousness, extraversion, and openness were reliably related to PEB [4]. Soutter et al. also found that Honesty-Humility, the trait that distinguishes the HEXACO model [5] from the FFM, was the strongest predictor of PEB (*r* = 0.25), although the relationship between PEB and openness was not much weaker (*r* = 0.21). Correlations between PEB and agreeableness, conscientiousness, and extraversion were 0.10 to 0.11. 

Although these results informed our study, there are some important differences between our approach and the approach taken in the research summarized in this meta-analysis. First, in the present study, we examined relationships between personality and climate change mitigation behavior, not the broader construct of pro-environmental behavior. Although CCBs are part of PEB, they are not the same. For example, sorting trash is a PEB, but it is not truly a CCB. Second, we examined relationships between CCB and personality using regression analyses which took into account relationships between personality factors. Previous research has relied primarily on zero-order correlations.

### 1.2. The Role of Neuroticism

Although Soutter et al.’s meta-analysis did not find that neuroticism was reliably related to PEB, we had reasons to believe that it would be positively related to CCB. The rationale for this expectation was the Extended Parallel Process Model of Witte and colleagues [6,7]. The model proposes that fear can motivate people to take action to fight a threat. In terms of the FFM, although fear per se is not part of neuroticism, anxiety, which is positively related to fear, is an important component of neuroticism. Consistent with this logic, a meta-analysis by van Valkengoed and Steg [8] found that negative affect (which neuroticism represents) was one of the strongest predictors (positive) of engaging in climate change adaptation behavior. This conclusion was also reached by Brosch [9] in another meta-analysis.

### 1.3. Perceived Efficacy and Climate Change Mitigation Behavior

Van Valkengoed and Steg’s meta-analysis also found that self-efficacy and outcome efficacy were both positively related to the frequency of adaptive behaviors. Adaptive behaviors refer to how people cope with or adapt to climate change more than they refer to attempts to mitigate climate change. The measures of efficacy in the studies they included in their meta-analysis tended to be behavior-specific. For example, Akompab et al. [10] found that the perceived benefits of certain actions to adapt to a heat wave were related to whether people engaged in those behaviors. Such relationships are consistent with a large body of research that examined Bandura’s model of behavioral change [11]. People are more likely to do things that produce the desired outcome than they are to do things that are less likely to produce desired outcomes.

Moreover, as noted by Bandura [12], self-efficacy should not be considered a general trait or disposition, “All too often, this belief system is treated as though it is a generalized trait. In fact, people differ in their efficacy, not only across different domains of functioning but even across various facets within an activity domain. Consequently, there is no single all-purpose measure of self-efficacy with a single validity coefficient.” In the studies reviewed by Van Valkengoed and Steg, efficacy was measured in terms of specific behaviors as recommended by Bandura.

### 1.4. Perceived Efficacy as a Mediator between Personality and Climate Change Mitigation Behavior

There is a large body of research that does not involve climate change mitigation behavior that supports the contention that efficacy beliefs mediate relationships between personality and performance [13]. Although this mediation tends to be incomplete, i.e., it does not account fully for relationships between the predictor and the outcome, the mediation tends to be meaningful. Complementing this, there are a growing number of studies involving various domains of climate change that have found that both outcome efficacy (also called response efficacy) and self-efficacy mediate relationships between some type of motive or disposition and behavior. For example, Demuth et al. [14] found that both response efficacy and self-efficacy mediated relationships between hurricane experience and hurricane preparedness. Along the same lines, Lefevre et al. [15] found that the perceived effectiveness of heat protection behaviors mediated relationships between knowledge about recommendations to cope with heat and how people coped with heat.

### 1.5. The Present Studies

To our knowledge, no study has examined if relationships between personality and climate change mitigation behavior are mediated by the perceived efficacy of mitigation behaviors. For example, none of the studies included in the meta-analysis of van Valkengoed and Steg [8] examined the types of common, everyday mitigation behaviors in which people can engage. Virtually all of the studies van Valkengoed and Steg included in their meta-analysis concerned mitigation behaviors for floods, heat waves, and other catastrophic events. Admittedly, floods and heat waves are the results of climate change, but van Valkengoed and Steg focused more on mitigating the effects of these climate-related disasters than on mitigating climate change itself, the root cause of these disasters.

The present studies were designed to complement the existing research on relationships between personality and climate change behavior by filling this gap. We conducted two studies. First, we examined the relationships between personality and climate change mitigation behavior. In the second study, we examined these same relationships and examined the extent to which the perceived efficacy of these behaviors mediated relationships between personality and climate change mitigation behavior.

Based on the meta-analysis of Souter et al. [4], we expected that self-reported climate change mitigation behavior (CCB) would be positively related to agreeableness, conscientiousness, extraversion, and openness. Noting this, the relationships Souter et al. found between PEB and agreeableness, conscientiousness, and extraversion were not very strong (approximately *r* = 0.10). It is difficult to find such relationships even with large samples. Based on the meta-analyses of van Valkengoed and Steg [8] and Brosch [9], we also expected that self-reported climate change mitigation behavior would be positively related to neuroticism.

Overall, we expected that the perceived effectiveness (efficacy) of CCB would mediate relationships between personality and CCB. Given the relative lack of theory and research about how such mediational relationships might vary across different combinations of personality and CCB, we did not have a basis for making predictions about how mediation might vary across different combinations of CCB and personality.

All data are available via the OSF repository: https://osf.io/79myt/?view_only=3ffd620f499f403fb1dcf7077cc17696 (accessed on 9 February 2023).

## 2. Study 1

### 2.1. Participants 

Participants were undergraduate students at a US university who were taking an introductory psychology course. They participated in partial fulfillment of a course requirement (*n* = 1089; *M*_age_ = 19.0, *SD* = 1.14; 687 women). Participants had the right to refuse to answer any question without penalty. Data were collected in the 2019–2020 academic year.

### 2.2. Measures

Participants completed the BFI-2, a measure of the Big Five model of personality [16]. They responded using the following 5-point scale: Disagree strongly, Disagree a little, Neither agree nor disagree, Agree a little, Agree strongly. Participants described how often they engaged in climate change mitigation behavior in terms of five behaviors. I limited my consumption of red meat. I limited my purchases to limit consumerism. I made others aware of the negative consequences of climate change. I signed a petition or/and participated in a demonstration about stopping climate change. I took various other actions related to stopping climate change. They responded using a 6-point scale with the following labels: Never; Not that often, less than once per week; Somewhat often, perhaps once a week; Often, a few times per week; Very often, 4–5 days per week; and Almost every day. 

### 2.3. Results

#### 2.3.1. Descriptive Statistics and Zero-Order Correlations between Measures 

The analyses for this study were conducted using SPSS, v. 24. Descriptive statistics, including reliabilities for the scales of the BFI-2 and zero-order correlations between measures, are presented in Table 1. According to the guidelines proposed by Shrout [17], four of five scales of the BFI-2 had substantial reliability (0.80 or greater), and one scale, agreeableness, had close to substantial reliability (0.79). 

#### 2.3.2. Relationships between Personality and Climate Change Mitigation Behavior

Relationships between personality and climate change mitigation behavior were examined in a series of regression analyses in which individual CCBs were regressed on the scores of the BFI-2. The results of these analyses are summarized in Table 2. As expected, openness was positively related to all CCBs. In addition, as expected, extraversion was positively related to CCBs that involved other people, i.e., making others aware, signing petitions, and attending demonstrations and meetings. Neuroticism was positively related to limiting the consumption of meat, making others aware, signing petitions, attending demonstrations and meetings, and taking other actions. Agreeableness was positively related to limiting the consumption of meat and taking other actions.

## 3. Study 2

### 3.1. Participants 

Participants were undergraduate students at a US university who were taking an introductory psychology course. They participated in partial fulfillment of a course requirement (*n* = 1688; *M*_age_ = 19.0, *SD* = 1.38; 1006 women). Participants had the right to refuse to answer any question without penalty. Data were collected in the 2020–2021 and 2021–2022 academic years.

### 3.2. Measures

Participants completed the same measures as in Study 1. They also described how often they engaged in two additional climate change mitigation behaviors: I limited using a car, and I reduced my consumption of electricity. In addition, participants indicated how effective they thought each CCB was by answering the following question: “How much do you think the following actions can (or could) help to prevent climate change?” They responded using a five-point scale labeled: Not at all, A little, Moderately, A lot, Very much. Given the lack of specificity of the CCB “I took other actions,” the perceived effectiveness of this CCB was not measured.

### 3.3. Results

#### 3.3.1. Descriptive Statistics and Zero-Order Correlations between Measures 

Descriptive statistics, including reliabilities for the scales of the BFI-2 and zero-order correlations between measures, are presented in Table 3. According to the guidelines proposed by Shrout [17] and similar to the results of Study 1, four of five scales of the BFI-2 had substantial reliability (0.80 or greater), and one scale, agreeableness, had close to substantial reliability (0.78). These analyses and the multiple regression analyses were conducted using SPSS, v. 24.

#### 3.3.2. Relationships between Personality and Climate Change Mitigation Behavior and Perceived Effectiveness of Mitigation Behavior 

Relationships between personality and climate change mitigation behavior were examined in a series of regression analyses the same as those used in Study 1. Individual CCBs were regressed on the scores of the BFI-2. The results of these analyses are summarized in Table 4. 

Similar to the results of Study 1, extraversion was positively related to making others aware, attending demonstrations/meetings, and signing petitions. Extraversion was also positively related to taking other actions. Similar to the results of Study 1, agreeableness was positively related to limiting the consumption of meat. Agreeableness was also positively related to limiting purchases and limiting the use of a car.

Similar to the results of Study 1, conscientiousness was positively related to purchases. Unlike Study 1, conscientiousness was positively related to limiting the consumption of meat, attending demonstrations/meetings, and signing petitions, and was positively related to the two added CCBs, limiting the use of a car and limiting the use of electricity.

Similar to the results of Study 1, neuroticism was positively related to limiting the consumption of meat, making others aware, attending demonstrations/meetings and signing petitions, and other actions. In addition, neuroticism was positively related to limiting purchases and to the two added CCBs, limiting the use of a car and limiting the use of electricity. Similar to Study 1, openness was positively related to all CCBs.

#### 3.3.3. Relationships between Personality and Perceived Effectiveness of Mitigation Behavior 

Relationships between personality and the perceived effectiveness of climate change mitigation behavior were examined in a series of regression analyses, the same as those used to examine relationships between personality and climate change mitigation. The most noteworthy result of these analyses is that extraversion was not related to the perceived effectiveness of any CCB. In contrast, all other factors were positively related to the perceived effectiveness of all CCBs, except for the relationship between agreeableness and the perceived effectiveness of limiting purchases. The results of these analyses are summarized in Table 5.

#### 3.3.4. Perceived Effectiveness as a Mediator of Relationships between Personality and Climate Change Mitigation Behavior 

We examined the perceived effectiveness of CCB-mediated relationships between personality and CC using a series of PROCESS models written for SPSS [18]. For each score of the BFI-2 that was significantly related to a CCB and that was significantly related to the perceived effectiveness of the same CCB, we examined the extent to which the perceived effectiveness of the CCB mediated the relationship between scores on that factor and self-reported CCB. 

These analyses used Model 4 of the PROCESS system. A CCB was the outcome, and a score on a factor of the BFI-2 was the predictor. The mediator was the perceived effectiveness of the CCB, which was the outcome, and scores on the other four factors were covariates. For example, openness was significantly (positively) related to limiting the consumption of meat and to the perceived effectiveness of limiting the consumption of meat (also positively). This met the requirements for mediation. Therefore, in the mediational analysis, self-reported limitation of the consumption of meat was the outcome, openness was the predictor, the perceived effectiveness of limiting the consumption of meat was the mediator, and extraversion, agreeableness, conscientiousness, and neuroticism were covariates. Note that we did not test for mediation for extraversion because relationships between extraversion and the perceived effectiveness of CCBs were not significant, which is required for mediation to exist.

The results of these analyses are summarized in Table 6. The table contains the total effect, the direct effect, and the indirect effect for each predictor for each CCB. The analyses used bootstrapping with 5000 resamples. As can be seen from the results presented in Table 6, the perceived effectiveness of CCB-mediated relationships between personality and self-reported CCB in all cases. In a majority of cases, the indirect effect accounted for over 25% of the total effect. Moreover, the direct effect was not significant (at the 0.05 level) in the analyses of relationships between agreeableness and limiting the use of a car, conscientiousness and limiting purchases and limiting electricity, neuroticism and attending demonstrations/signing petitions, and openness and attending demonstrations/signing petitions.

## 4. Discussion

### 4.1. Relationships between Personality and Climate Change Mitigation Behavior

As expected, self-reported climate change mitigation behavior was positively related to numerous factors of the FFM. In both studies, extraversion was positively related to CCBs that involved doing something with other people, i.e., making others aware and attending demonstrations/meetings, and signing petitions. Such relationships are consistent with the fact that social activity is one of the defining characteristics of extraversion. 

In study 1, neuroticism was positively related to limiting the consumption of meat, making others aware, participating in demonstrations, and taking other actions, four of the five behaviors we measured. In Study 2, neuroticism was positively related to all CCBs. Such relationships are consistent with the model we presented in the introduction that fear and anxiety can motivate people to take action in the face of a threat.

In both Studies 1 and 2, openness was positively related to all CCBs. Such positive relationships are consistent with the results of previous research [4] and with the conceptualization of openness to include willingness to consider new possibilities, e.g., that the climate is changing and something needs to be done about it.

In Study 1, conscientiousness was positively related only to limiting purchases, whereas in Study 2, it was positively related to five of seven CCBs. To the extent that mitigation behavior can be seen as appropriate and normative, such positive relationships reflect the adherence to norms, “to do the right thing,” which is part of conscientiousness. 

In study 1, agreeableness was related to two CCBs, and in Study 2, it was related to three. At first glance, this might seem to be contrary to the fact that agreeableness is often considered to be a defining characteristic of prosociality [19]. Fighting climate change is prosocial, so agreeableness should be positively related to climate change mitigation behavior because agreeableness is prosocial. Nevertheless, as argued (and demonstrated) by Nezlek [2], interpersonal prosociality, which has been the focus of research examining relationships between prosociality and agreeableness, is distinct from ideological prosociality, and climate change mitigation is ideologically prosocial. Agreeableness refers to being pleasant to individuals, which does not include advocating prosocial policies or taking prosocial actions to benefit society as a whole.

### 4.2. Perceived Effectiveness as a Mediator of Relationships between Personality and Climate Change Mitigation Behavior

As expected, the perceived effectiveness of CCBs mediated all of the relationships when the conditions for mediation were met, i.e., there was a significant relationship between a personality factor and a CCB, and there was a significant relationship between a personality factor and the perceived effectiveness of a CCB. To our knowledge, this is the first study to demonstrate that perceived effectiveness can mediate relationships between personality and CCBs (or pro-environmental behavior defined more broadly). As such, our findings add meaningfully to the existing body of research on understanding climate change mitigation behavior. 

Substantively, our results suggest that for most personality traits, relationships between these traits and mitigation behavior are explained partially by relationships between traits and the perceived efficacy of behaviors. Although the indirect effects we found accounted for at least 25% of the total effect in most analyses, this still leaves much of the total effects due to direct effects between traits and behaviors. In terms of possible causality, our results suggest a causal sequence of personality traits to perceptions of efficacy to climate change mitigation behavior. We discuss this topic in the section below. 

An interesting exception to this pattern was extraversion. Although extraversion was significantly related to three CCBs in Studies 1 and 2, extraversion was not significantly related to the perceived effectiveness of any CCB in Study 2, which meant no mediation was possible. These results suggest that the influence extraversion has on CCBs that involve contact with other people (making others aware and participating in demonstrations) is direct. Extraversion is positively related to social activity, and this seems to include social activities involving climate change mitigation.

### 4.3. Moderation vs. Mediation

We examined mediation to understand more about why personality might be related to CCB. Alternatively, we could have used moderation to understand more about when or under what circumstances personality is related to CCB. Given the extensive body of research that has examined how perceived efficacy mediates relationships between some types of predictors (motivation, experience, etc.) and some types of behaviors, we focused on mediation. Nevertheless, we repeated the analyses we described with mediators and moderators and found only two significant moderating effects (*p* ≤ 0.05). The perceived efficacy of limiting meat and moderated relationships between limiting meat and agreeableness and openness such that these relationships were stronger for people with stronger efficacy beliefs than for those with weaker efficacy beliefs. The relative lack of moderating relationships suggests that efficacy is best thought of as a mediator between personality and CCB, not as a moderator of the relationship between personality and CCB. 

### 4.4. Effect Sizes

Collectively, the five factors of the FFM accounted for approximately 2–7% of the variance in mitigation behavior. This varied somewhat across CCBs, although the relationships tended to be stronger in Study 2 than in Study 1, perhaps due to the larger sample in Study 2. Regardless, such effect sizes may raise questions for some about the importance or meaning of the present findings.

We believe that the present effect sizes need to be understood within the following context. It is likely that engaging in CCBs is influenced by multiple factors, of which personality is only one. Moreover, small effects can be meaningful when they accumulate [20], and this is likely to be the case for outcomes related to personality, which is a constant in people’s lives. In addition, in the present study, the levels of measurement of personality and CCB were different. Personality was measured without regard to time, more or less referring to a permanent state, whereas CCB was measured over a short period of time (two weeks). Such differences in the level of measurement can lead to an underestimation of the strength of relationships between measures of constructs. If we had measured CCB over a longer period of time, the relationships between personality and CCB might have been stronger. See Epstein [21] for a discussion of this issue and a demonstration of such a possibility.

### 4.5. Mediation and Causality

Implicit in our discussion of mediation has been a causal sequence in which personality leads to perceptions of the effectiveness of CCB which in turn lead to CCB. Moreover, such a causal sequence has been assumed in much of the research that has examined perceived efficacy as a mediator between some types of motive/disposition and some types of behavior. For example, Caprara et al. [22] discussed how “basic traits are relatively unconditional, broad dispositions,” whereas “self-efficacy is a knowledge structure (i.e., a set self-related beliefs) operating at an intermediate level between broad dispositions and specific behavior”.

Although such a causal sequence may make sense theoretically, the results of a single-occasion, cross-sectional study such as ours do not provide a basis to support claims of causality. The mediation we found could be considered necessary support for the proposed causal sequence, but the mediation we found cannot be considered sufficient support for the proposed causal sequence. This requires a study that examines changes across time.

### 4.6. Sample Characteristics

Participants were US collegians, and it is not certain how this might have influenced the results. The constructs we measured were appropriate for our sample, and the relationships we found were consistent with the results of previous research. Moreover, our participants were “emerging adults,” a group for which climate change is particularly relevant [23]. Nevertheless, it cannot be assumed that the relationships we found would occur in a different sample (e.g., adults in a country other than the US) or with different measures. In fact, we found some inconsistencies between the two samples we studied. Moreover, although we believe that our measures of mitigation behavior represent a core set of mitigation behaviors, other behaviors could be studied.

## 5. Conclusions

The implications of the zero-order relationships we found between personality and climate change mitigation for practice are not clear. It seems unlikely that programs or interventions can be designed to change personality in ways that would increase mitigation behavior. Nevertheless, understanding how personality is related to climate change mitigation behavior and the perceived effectiveness of mitigation behavior may help people who develop interventions to increase mitigation behavior in some way.

Our results regarding perceived effectiveness may have more direct implications in practice. The present results suggest that the perceived efficacy of climate change mitigation behavior is a proximal cause of mitigation behavior. Assuming this is the case, interventions designed to increase people’s recognition of the effectiveness of mitigation behaviors would seem to hold promise. Put simply; people are much more likely to do something if they believe that doing this will accomplish some type of goal than if they do not believe it will. The present results suggest some ways that such interventions might be tailored to appeal to people with different personalities, but the overall conclusion cuts across such differences.

We recognize that the present results are preliminary. Establishing the validity of the relationships we have described requires replication using different samples and studies across time. Nevertheless, we believe that the present results are valuable in and of themselves and can provide a starting point for future research.

## Figures and Tables

**Table 1:** Descriptive statistics and zero-order correlations between measures.

	*M*	*SD*	E	A	C	N	O	Meat	Buy	Aware	Demo
Extraversion (E)	3.29	0.78	0.88								
Agreeableness (A)	3.79	0.60	**0.12**	0.79							
Conscientiousness (C)	3.53	0.71	**0.20**	**0.29**	0.86						
Negative Emotionality (N)	2.98	0.84	**−0.30**	**−0.27**	**−0.25**	0.89					
Open-Mindedness (O)	3.83	0.65	**0.22**	**0.13**	**0.07**	−0.01	0.83				
Limited consumption of red meat (Meat)	2.87	1.95	−0.01	**0.09**	0.05	**0.10**	**0.10**				
Limited purchases (Buy)	2.60	1.56	−0.03	0.05	**0.07**	0.03	**0.16**	**0.47**			
Made others aware (Aware)	2.17	1.25	**0.12**	0.03	0.05	**0.06**	**0.12**	**0.35**	**0.43**		
Petitions, demonstrations, and meetings (Demo)	1.48	0.88	**0.07**	0.03	-0.01	0.04	**0.09**	**0.26**	**0.34**	**0.46**	
Took other actions	2.43	1.45	0.05	**0.09**	0.04	0.03	**0.12**	**0.43**	**0.47**	**0.53**	**0.38**

Note: Cronbach’s alpha for each personality scale is on the diagonal. Significant correlations are in **bold**. *r* > |0.06| *p* < 0.05; *r* > |0.08| *p* < 0.01.

**Table 2:** Study 1: Relationships between personality and climate change mitigation behavior.

Behavior	E	A	C	N	O	*R*	*F*
Limit meat	−0.007	0.102 **	0.052	0.142 **	0.081 **	0.185	7.65
Limit purchase	−0.073 *	0.010	0.053 *	0.026	0.165 **	0.185	7.67
Make others aware	0.114 **	0.014	0.020	0.117 **	0.094 **	0.187	7.86
Demo/petition/meeting	0.075 *	0.042	−0.027	0.066 *	0.071 *	0.126	3.52
Other action	0.041	0.089 **	0.020	0.077 *	0.100 **	0.161	5.78

Note: See Table 1 for labels for measures. For all models, *df*s were 5,1083. * *p* < 0.05; ** *p* < 0.01. All *F*-ratios were significant at *p* < 0.01.

**Table 3:** Study 2 Descriptive statistics and zero-order correlations between measures.

	*M*	*SD*	Sample	E	A	C	N	O	Meat	Buy	Aware	Demo	Car	Elec	Other	E-Meat	E-Buy	E-Aware	E-Demo	E-Car
Extraversion ©	3.23	0.77	1688	0.87																
Agreeableness (A)	3.75	0.59	1688	**0.10**	0.78															
Conscientiousne© (C)	3.55	0.70	1688	**0.22**	**0.25**	0.85														
Negative Emotionality (N)	3.04	0.85	1688	**−0.29**	**−0.22**	**−0.28**	0.90													
Open-Mindedness (O)	3.87	0.65	1688	**0.18**	**0.21**	**0.09**	**0.06**	0.83												
Limited consumption of red meat (Meat)	2.82	1.99	1551	0.02	**0.13**	**0.07**	**0.15**	**0.19**												
Limited purchases (Buy)	2.81	1.73	1543	0.01	**0.10**	**0.05**	**0.12**	**0.23**	**0.53**											
Made others aware (Aware)	2.16	1.30	1641	**0.11**	0.03	0.00	**0.10**	**0.16**	**0.40**	**0.44**										
Petitions, demonstrations, and meetings (Demo)	1.73	1.15	1640	**0.09**	0.01	**−0.07**	**0.09**	**0.10**	**0.33**	**0.36**	**0.65**									
Limited use of car (Car)	3.40	2.05	1389	−0.04	**0.08**	**0.07**	**0.13**	**0.18**	**0.40**	**0.50**	**0.34**	**0.29**								
Limited electricity (Elec).	3.02	1.79	1609	0.03	0.04	**0.07**	**0.09**	**0.13**	**0.34**	**0.45**	**0.35**	**0.29**	**0.46**							
Took other actions (Other)	2.45	1.55	1587	**0.10**	0.04	**0.06**	**0.05**	**0.19**	**0.42**	**0.48**	**0.53**	**0.46**	**0.41**	**0.54**						
Effective meat (E-Meat)	2.99	1.24	1674	0.02	**0.07**	0.04	**0.13**	**0.17**	**0.46**	**0.34**	**0.31**	**0.23**	**0.27**	**0.24**	**0.30**					
Effective buy (E-Buy)	3.11	1.20	1671	−0.01	**0.06**	**0.06**	**0.16**	**0.18**	**0.36**	**0.44**	**0.31**	**0.24**	**0.31**	**0.29**	**0.31**	**0.67**				
Effective aware (E-Aware)	3.34	1.17	1688	0.02	**0.11**	**0.10**	**0.10**	**0.14**	**0.24**	**0.22**	**0.32**	**0.24**	**0.24**	**0.22**	**0.25**	**0.41**	**0.48**			
Effective demo (E-Demo)	2.84	1.18	1684	0.00	**0.14**	**0.07**	**0.13**	**0.15**	**0.28**	**0.25**	**0.32**	**0.32**	**0.25**	**0.22**	**0.26**	**0.37**	**0.41**	**0.63**		
Effective car (E-Car)	3.62	1.13	1685	−0.02	**0.09**	**0.12**	**0.12**	**0.12**	**0.20**	**0.23**	**0.17**	**0.13**	**0.29**	**0.22**	**0.20**	**0.43**	**0.55**	**0.52**	**0.46**	
Effective electricity (E-Elec)	3.47	1.16	1685	0.00	**0.09**	**0.13**	**0.11**	**0.10**	**0.19**	**0.24**	**0.19**	**0.16**	**0.27**	**0.29**	**0.23**	**0.44**	**0.54**	**0.51**	**0.46**	**0.81**

Note: Cronbach’s alpha for each personality scale is on the diagonal. Significant correlations are in **bold**. *r* > |0.05| *p* < 0.05; *r* > |0.07| *p* < 0.01, except for *n* = 1389, *r* > |0.05| *p* < 0.05.

**Table 4:** Relationships between personality and climate change mitigation behavior: Study 2.

Behavior	E	A	C	N	O	*R*	*F*	*df*
Limit meat	0.023	0.118 **	0.078 **	0.200 **	0.137 **	0.277	25.69	1545
Limit purchase	−0.003	0.071 **	0.056 *	0.133 **	0.197 **	0.263	22.90	1537
Make others aware	0.128 **	0.019	−0.007	0.136 **	0.121 **	0.218	16.36	1635
Demo/petition/meeting	0.115 **	0.025	−0.075 **	0.105 **	0.078 **	0.181	11.04	1634
Limit car	−0.048	0.058 *	0.091 **	0.141 **	0.158 **	0.242	17.17	1383
Limit electricity	0.020	0.016	0.086 **	0.113 **	0.111 **	0.179	10.60	1603
Other action	0.087 **	0.005	0.042	0.078 **	0.167 **	0.218	15.79	1581

Note: See Table 1 and Table 3 for labels for measures. * *p* < 0.05; ** *p* < 0.01. All *F*-ratios were significant at *p* < 0.01. For all models, the numerator *df* was 5. Denominator *df*s are presented in the column labeled df.

**Table 5:** Relationships between personality and perceived effectiveness of climate change mitigation behavior.

Behavior	E	A	C	N	O	*R*	*F*	*df*
Limit meat	0.016	0.062 *	0.055 *	0.150 **	0.140 **	0.223	17.49	1668
Limit purchase	−0.011	0.044	0.083 **	0.182 **	0.156 **	0.255	23.18	1665
Make others aware	0.016	0.095 **	0.103 **	0.146 **	0.103 **	0.224	17.71	1682
Demo/petition/meeting	0.006	0.136 **	0.075 **	0.175 **	0.099 **	0.247	14.79	1678
Limit car	−0.026	0.069 **	0.148 **	0.162 **	0.089 **	0.234	19.40	1679
Limit electricity	−0.004	0.078 **	0.148 **	0.167 **	0.065 **	0.227	18.29	1679

Note: See Table 1 and Table 3 for labels for measures. * *p* < 0.05; ** *p* < 0.01. All *F*-ratios were significant at *p* < 0.01. For all models, the numerator *df* was 5. Denominator *df*s are presented in the column labeled *df*.

**Table 6:** Perceived effectiveness as a mediator between personality and mitigation behavior.

	Sample	Effect	A	CI	%	C	CI	%	N	CI	%	O	CI	%
Limited consumption	1538	total	0.397 **			0.233 **			0.456 **			0.431 **		
of red meat		direct	0.304 **			0.161 **			0.298 **			0.231 **		
		indirect	0.093	0.016/0.073	23	0.072	0.006/0.142	31	0.159	0.104/0.215	35	0.200	0.134/0.270	46
Limited purchases	1534	total				0.143 **			0.260 **			0.527 **		
		direct				0.053 ^ns^			0.104 *			0.344 **		
		indirect				0.089	0.034/0.147	62	0.156	0.112/0.203	60	0.183	0.125/0.246	35
Made others aware	1641	total							0.207 **			0.245 **		
		direct							0.137 **			0.103 **		
		indirect							0.070	0.045/0.097	34	0.062	0.030/0.094	25
Petitions, demonstrations,	1636	total				−0.121 **			0.140 **			0.139 **		
and meetings		direct				−0.161 *			0.067 ^ns^			0.082 ^ns^		
		indirect				0.039	0.010/0.068		0.073	0.049/0.101	52	0.057	0.029/0.087	41
Limited use car	1386	total	0.194 *			0.254 **			0.328 **			0.496 **		
		direct	0.141 ^ns^			0.166 *			0.231 **			0.409 *		
		indirect	0.052	0.004/0.104	27	0.088	0.047/0.134	35	0.097	0.060/0.136	30	0.087	0.044/0.134	18
Limited electricity	1608	total				0.221 **			0.234 **			0.309 **		
		direct				0.123 ^ns^			0.141 *			0.259 **		
		indirect				0.098	0.061/0.140	44	0.091	0.061/0.129	39	0.050	0.013/0.090	16

Note: Columns labeled CI contain 95% confidence intervals for indirect effects. Columns labeled % contain the percent of the total effect accounted for by the indirect effect. For total and direct effects, * *p* < 0.05; ** *p* < 0.01.

## Data Availability

As noted in the text, all data and materials are available via the Open Science Foundation https://osf.io/79myt/?view_only=3ffd620f499f403fb1dcf7077cc17696 (accessed on 9 February 2023).
